# Supplementary material for: Dynamic pricing strategies towards strategic consumers under demand learning
Source: PLoS One. 2026 Jan 2;21(1):e0340105. doi: 10.1371/journal.pone.0340105 (PMC12758830; doi:10.1371/journal.pone.0340105)
Supplement: S1 Appendix — (DOCX) [file pone.0340105.s001.docx]

**Appendix to the article “Dynamic Pricing Strategies towards Strategic Consumers under Demand Learning”**

# Appendix A：Proofs of Statements

**Proof of Lemma 1**

The prior Gamma distribution of the market size $\Lambda$ expresses $f\left( \Lambda=\lambda\right)=\frac{\lambda^{\alpha-1}\beta^{\alpha}e^{-\beta\lambda}}{\Gamma(\alpha)}$, where $\Gamma\left( \alpha\right)=\int_{0}^{+\infty} e^{-x}x^{\alpha-1}dx$. Given $\Lambda=\lambda$ and price response function $\Psi_{1}\left( p_{1} \right)$, the conditional probability distribution of the first-stage demand is a Poisson distribution with mean $\lambda\Psi_{1}\left( p_{1} \right)$, specifically:

$$\begin{aligned} P\left[ D_{1}=x | \lambda\Psi_{1}\left( p_{1} \right) \right]=\frac{\left[ \lambda\Psi_{1}\left( p_{1} \right) \right]^{x}e^{-\lambda\Psi_{1}\left( p_{1} \right)}}{x!}\#\left( A1 \right) \end{aligned}$$

Combining Eq. ([A1](#A1)) and the prior Gamma distribution of $\Lambda$, the unconditional prior distribution of $D_{1}$ is obtained as:

$$\begin{aligned} \begin{aligned} P\left( D_{1}=x \right)=&\int_{0}^{\infty} P\left[ D_{1}=x | \lambda\Psi_{1}\left( p_{1} \right) \right]f\left( \lambda\right)d\lambda\# \\ =&\int_{0}^{\infty} \frac{\left[ \lambda\Psi_{1}\left( p_{1} \right) \right]^{x}e^{-\lambda\Psi_{1}\left( p_{1} \right)}}{x!}\cdot\frac{\lambda^{\alpha-1}\beta^{\alpha}e^{-\beta\lambda}}{\Gamma\left( \alpha\right)}d\lambda\\ =&\frac{\Gamma\left( \alpha+x \right)}{\Gamma\left( x+1 \right)\Gamma\left( \alpha\right)}\frac{\beta^{\alpha}}{\left( \beta+1 \right)^{\alpha+x}} \\ =&\binom{\alpha+x-1}{x}\left( \frac{\beta}{\beta+\Psi_{1}\left( p_{1} \right)} \right)^{\alpha}\left( \frac{\Psi_{1}\left( p_{1} \right)}{\beta+\Psi_{1}\left( p_{1} \right)} \right)^{x} \end{aligned}\left( A2 \right) \end{aligned}$$

It is a negative binomial distribution with parameters $\left( \alpha,\frac{\beta}{\beta+\Psi_{1}\left( p_{1} \right)} \right)$, whose expectation is $E\left( D_{1} \right)=\Psi_{1}\left( p_{1} \right)\frac{\alpha}{\beta}$.

Observing the real sales $x_{1}$in the first stage, the seller obtains the posterior distribution of $\Lambda$ according to Bayesian rules as follows:

$$\begin{aligned} f\left( \Lambda=\lambda|x_{1} \right)=\frac{P\left[ D_{1}=x_{1} | \lambda\Psi_{1}\left( p_{1} \right) \right]f\left( \lambda\right)}{\int_{0}^{\infty} P\left[ D_{1}=x_{1} | \lambda\Psi_{1}\left( p_{1} \right) \right]f\left( \lambda\right)d\lambda}=\frac{\lambda^{\left( \alpha+x_{1}-1 \right)}\left( \beta+\Psi_{1}\left( p_{1} \right) \right)^{\left( \alpha+x_{1} \right)}e^{-\lambda\left( \beta+\Psi_{1}\left( p_{1} \right) \right)}}{\Gamma\left( \alpha+x_{1} \right)}\#\left( A3 \right) \end{aligned}$$

which is also a Gamma distribution with parameters $\left( \alpha+x_{1},\beta+\Psi_{1}\left( p_{1} \right) \right)$.

We regard the updated $f\left( \Lambda=\lambda|x_{1} \right)$ by demand learning as the prior distribution of market size $\tilde{\Lambda}$ in the second stage, donating as $f\left( \tilde{\Lambda}=\tilde{\lambda} \right)$. When price response function $\Psi_{2}\left( p_{2} \right)$ in the second stage is deterministic, the updated unconditional distribution $D_{2}$ is:

$$\begin{aligned} \begin{aligned} P\left( D_{2}=x \right)=&\int_{0}^{\infty} P\left[ D_{2}=x | \Psi_{2}\left( p_{2} \right)\tilde{\lambda} \right]f\left( \tilde{\lambda} \right)d\tilde{\lambda}\# \\ =&\int_{0}^{\infty} \frac{\left[ \Psi_{2}\left( p_{2} \right)\tilde{\lambda} \right]^{x}e^{-\Psi_{2}\left( p_{2} \right)\tilde{\lambda}}}{x!}\cdot\frac{\lambda^{\left( \alpha+x_{1}-1 \right)}\left( \beta+\Psi_{1}\left( p_{1} \right) \right)^{\left( \alpha+x_{1} \right)}e^{-\lambda\left( \beta+\Psi_{1}\left( p_{1} \right) \right)}}{\Gamma\left( \alpha+x_{1} \right)}d\tilde{\lambda} \\ =&\binom{{\alpha+x}_{1}+x-1}{x}\left( \frac{\beta+\Psi_{1}\left( p_{1} \right)}{\beta+\Psi_{1}\left( p_{1} \right)+\Psi_{2}\left( p_{2} \right)} \right)^{\left( {\alpha+x}_{1} \right)}\left( \frac{\Psi_{2}\left( p_{2} \right)}{\beta+\Psi_{1}\left( p_{1} \right)+\Psi_{2}\left( p_{2} \right)} \right)^{x} \end{aligned}\#\left( A4 \right) \end{aligned}$$

Similarly, this is also a negative binomial distribution with parameters $\left( {\alpha+x}_{1},\frac{\beta+\Psi_{1}\left( p_{1} \right)}{\beta+\Psi_{1}\left( p_{1} \right)+\Psi_{2}\left( p_{2} \right)} \right)$ and its mean $E\left( D_{2} \right)=\frac{({\alpha+x}_{1})\Psi_{2}\left( p_{2} \right)}{\beta+\Psi_{1}\left( p_{1} \right)}$.

Lemma 1 is proved.

**Proof of** **Theorem 1**

There are four dynamic pricing strategies for the seller: dynamic pricing without price guarantee and demand learning (Strategy $DN$); dynamic pricing without price guarantee and with demand learning (Strategy $DL$); dynamic pricing with price guarantee and without demand learning (Strategy $GN$); dynamic pricing with price guarantee and demand learning (Strategy $GL$). We represent these strategies as $K$, where $K=\{DN,DL,GN,GL\}$.

We first analyze the decision of a consumer (she) **in the first stage**. A consumer decides to buy a product in the first stage, the following two conditions must be met:

$$\begin{aligned} \left\{ \begin{aligned} &v-p_{1}^{K}\geq0 \\ &U_{1}^{K}\geq U_{2}^{K} \end{aligned} \right.\#\left( A5 \right) \end{aligned}$$

For $K=\{DN,DL\}$, the seller implements dynamic pricing without/with demand learning when price guarantee is offered. Given $Q, p_{1}^{K}$ and the belief of the second-stage price $\hat{p}_{2}^{K}\left( q \right), q=\left( Q-x_{1} \right)^{+}$, the expected surplus of a consumer with valuation $v$ at each stage is as follows.

$$\begin{aligned} U_{1}^{K}=f_{1}^{K}\left( Q \right)\cdot\left( v-p_{1}^{K} \right), U_{2}^{K}=\delta\sum_{x_{1}=0}^{Q} f_{2}^{K}\left( q \right)\cdot(v-\hat{p}_{2}^{K}(q))\cdot Pr(D_{1}^{K}=x_{1})\#\left( A6 \right) \end{aligned}$$

$f_{1}^{K}\left( Q \right)$, $q$ and $f_{2}^{K}\left( q \right)$ are correlated each other, but them are independent of a consumer's valuation $v$. When $v=0$, we have $U_{2}^{K}|_{v=0}\geq0>U_{1}^{K}|_{v=0}=-p_{1}^{K}f_{1}^{K}\left( Q \right)$, which means if the consumer's valuation $v=0$, the expected surplus in the first stage is less than that in the second stage. And when $v\in(0,1]$, there are two cases. One is that $U_{2}^{K}>U_{1}^{K}$ is strictly hold, so it is obviously optimal for the consumer to purchase in the second stage. In other words, the threshold adopted in the first stage is $\bar{v}_{1}^{K}=1$. The other case is that there are cross-points about the two functions within $v\in(0,1]$ (i.e., $U_{1}^{K}=U_{2}^{K}$). Because both two-stage expected surplus are linear functions of valuation $v$, and the inequality $f_{1}^{K}\left( Q \right)>\sum_{x_{1}=0}^{Q} f_{2}^{K}\left( q \right)\cdot Pr(D_{1}^{K}=x_{1})$ is strictly hold (see the appendix of [Aviv et al.](#AvivandZhang2019) ([2019](#AvivandZhang2019))), there is $\frac{\partial U_{1}^{K}}{\partial v}=f_{1}^{K}\left( Q \right)>\delta\sum_{x_{1=0}}^{Q} f_{2}^{K}\left( q \right)\cdot Pr(D_{1}^{K}=x_{1})=\frac{\partial U_{2}^{K}}{\partial v}$, that is, the two functions have a *unique* intersection $\bar{v}_{1}^{K}=v_{1}^{K}\left( p_{1}^{K} \right)$within $v\in(0,1]$, and $v_{1}^{K}\left( p_{1}^{K} \right)$ is the solution to $U_{1}^{K}=U_{2}^{K}$. When $v\geq v_{1}^{K}\left( p_{1}^{K} \right)$, the consumer chooses to buy in the first stage, otherwise she chooses to wait. Next, we consider to prove $v_{1}^{K}\left( p_{1}^{K} \right)\geq p_{1}^{K}$. When $v=p_{1}^{K}$, $U_{2}^{K}|_{v=p_{1}^{K}}=\delta\sum_{x_{1=0}}^{Q} f_{2}^{K}\left( q \right)\cdot(p_{1}^{K}-\hat{p}_{2}^{K}(q))\cdot Pr(D_{1}^{K}=x_{1})\geq0\geq U_{1}^{K}|_{v=p_{1}^{K}}=0$, as a result, the consumer chooses to wait until the second stage, and we have $p_{1}^{K}\leq v_{1}^{K}\left( p_{1}^{K} \right)$. Consequently, if the seller adopts strategy $K=\{DN,DL\}$, a consumer decides to purchase when $v\geq v_{1}^{K}\left( p_{1}^{K} \right)\in[p_{1}^{K},1]$.

For $K=\{GN,GL\}$, the seller chooses whether to implement demand learning in dynamic pricing with price guarantee. Given $Q, p_{1}^{K}$ and the belief of the second-stage price $\hat{p}_{2}^{K}\left( q \right), q=\left( Q-x_{1} \right)^{+}$, the expected utility surplus of the consumer at each stage is:

$$\begin{aligned} \left\{ \begin{aligned} &U_{1}^{K}=f_{1}^{K}\left( Q \right)\cdot\left[ v-p_{1}^{K}+\delta\sum_{x_{1}=0}^{Q} \left( p_{1}^{K}-\hat{p}_{2}^{K}\left( q \right) \right)^{+}\cdot\Pr\left( D_{1}^{K}=x_{1} \right) \right] \\ &U_{2}^{K}=\delta\sum_{x_{1}=0}^{Q} f_{2}^{K}\left( q \right)\cdot\left( v-\hat{p}_{2}^{K}\left( q \right) \right)\cdot\Pr\left( D_{1}^{K}=x_{1} \right) \end{aligned} \right.\#\left( A7 \right) \end{aligned}$$

Here we discuss two cases. One is that the consumer estimates there will be no markdown in the second stage (i.e., $p_{1}^{K}\leq\hat{p}_{2}^{K}\left( q \right)$), the expected utility in the first stage is simplified as $U_{1}^{K}=f_{1}^{K}\left( Q \right)\left( v-p_{1}^{K} \right)$ duo to without price difference compensation. Since $f_{1}^{K}\left( Q \right)>\sum_{x_{1}=0}^{Q} f_{2}^{K}\left( q \right)\cdot Pr(D_{1}^{K}=x_{1})$, the inequality $U_{1}^{K}=f_{1}^{K}\left( Q \right)\cdot\left( v-p_{1}^{K} \right)>\delta\sum_{x_{1}=0}^{Q} f_{2}^{K}\left( q \right)\cdot\left( v-\hat{p}_{2}^{K}\left( q \right) \right)\cdot Pr(D_{1}^{K}=x_{1})=U_{2}^{K}$ is strictly established, and we intuitively find that the consumer will purchase products in the first stage once the valuation is higher than $p_{1}^{K}$.

The other is that there is a markdown in the second stage (i.e., $p_{1}^{K}>\hat{p}_{2}^{K}\left( q \right)$), the expected utility of two stages are $U_{1}^{K}=f_{1}^{K}\left( Q \right)\cdot\left[ v-p_{1}^{K}+\delta\sum_{x_{1}=0}^{Q} \left( p_{1}^{K}-\hat{p}_{2}^{K}\left( q \right) \right)\cdot\Pr\left( D_{1}^{K}=x_{1} \right) \right]$, $U_{2}^{K}=\delta\sum_{x_{1}=0}^{Q} f_{2}^{K}\left( q \right)\cdot\left( v-\hat{p}_{2}^{K}\left( q \right) \right)\cdot\Pr\left( D_{1}^{K}=x_{1} \right)$, respectively. For indifferent purchase between two stages $U_{1}^{K}=U_{2}^{K}$, the solution is

$v=v_{1}^{K}\left( p_{1}^{K} \right)=\frac{f_{1}^{K}\left( Q \right)(1-\delta)p_{1}^{K}+\delta\left( f_{1}^{K}\left( Q \right)-\sum_{x_{1}=0}^{Q} f_{2}^{K}\left( q \right)\cdot\Pr\left( D_{1}^{K}=x_{1} \right) \right)\sum_{x_{1}=0}^{Q} \hat{p}_{2}^{K}\left( q \right)\cdot\Pr\left( D_{1}^{K}=x_{1} \right)}{f_{1}^{K}\left( Q \right)-\delta\sum_{x_{1}=0}^{Q} f_{2}^{K}\left( q \right)\cdot\Pr\left( D_{1}^{K}=x_{1} \right)}$. However, according to the inequalities [A5](#A5), the consumer chooses to purchase in the first stage must satisfy $v-p_{1}^{K}\geq0$. The difference $v_{1}^{K}\left( p_{1}^{K} \right)-p_{1}^{K}=-\frac{\delta\sum_{x_{1}=0}^{Q} \left( p_{1}^{K}-\hat{p}_{2}^{K}\left( q \right) \right)\cdot\Pr\left( D_{1}^{K}=x_{1} \right)\cdot\left( f_{1}^{K}\left( Q \right)-\sum_{x_{1}=0}^{Q} f_{2}^{K}\left( q \right)\cdot\Pr\left( D_{1}^{K}=x_{1} \right) \right)}{f_{1}^{K}\left( Q \right)-\delta\sum_{x_{1}=0}^{Q} f_{2}^{K}\left( q \right)\cdot\Pr\left( D_{1}^{K}=x_{1} \right)}$. Since $p_{1}^{K}-\hat{p}_{2}^{K}\left( q \right)>0$ and $f_{1}^{K}\left( Q \right)\geq\sum_{x_{1}=0}^{Q} f_{2}^{K}\left( q \right)\cdot\Pr\left( D_{1}^{K}=x_{1} \right)$, there is always $v_{1}^{K}\left( p_{1}^{K} \right)-p_{1}^{K}<0$, which means that only when the consumer's valuation is higher than $p_{1}^{K}$, she will purchase a product in the first stage.

Consequently, when the seller adopts strategy $K=\{GN,GL\}$, the threshold for a consumer to choose to purchase products in the first stage is $\bar{v}_{1}^{K}=p_{1}^{K}\leq1$, that is, consumers directly consider surplus in the first stage to make purchasing decisions, without considering the future price.

When a consumer waits to **the second stage**, her valuation is lower than the purchase threshold of the first stage $\bar{v}_{1}^{K}$. Besides, in such case, whether she purchases a product in the second stage depends on net utility of a product. If she leaves the market without buying anything, the net utility is zero. Consequently, there is an inequality $\delta\left( v-\hat{p}_{2}^{K}\left( q \right) \right)\geq0$ for purchase decisions in the second stage, that is $v\geq\bar{v}_{2}^{K}=\hat{p}_{2}^{K}(q)$*.*

The reference is as follows.

Aviv, Y., Wei, M.M., Zhang, F., 2019. Responsive Pricing of Fashion Products: The Effects of Demand Learning and Strategic Consumer Behavior. *Management Science* 65(7), 2982–3000.

Theorem 1 is proved.

**Proof of Proposition 1**

Given $K,Q, p_{1}^{K}$ and the belief of the second-stage price $\hat{p}_{2}^{K}(q)$*,* Theorem 1 has obtained an individual consumers' purchase threshold in the first stage. Because the consumers' belief of the second-stage price $\hat{p}_{2}^{K}(q)$ are formed by Rational Expectation Equilibrium and is unique, her purchase threshold in the first stage $\bar{v}_{1}^{K}$ is unique. Following to the proof of Proposition 1 in [Aviv and Pazgal](#AvivPazgal2008) ([2008](#AvivPazgal2008)), due to the specification of the information structure in our model, assessment of the expected surplus at each stage is the same for all consumers in the market. Therefore, the purchase strategy of the *other* consumers in the first stage is consistent with that of the individual consumer described in Theorem 1. Therefore, the unique purchase threshold $\bar{v}_{1}^{K}$ in the first stage is identical to all consumers.

The reference is as follows.

Aviv, Y., Pazgal, A., 2008. Optimal Pricing of Seasonal Products in the Presence of Forward-Looking Consumers. *Manufacturing & Service Operations Management* 10(3), 339-359.

Proposition 1 is proved.

**Proof of Theorem 2**

Given the optimal first-stage price $p_{1}^{DL*}$, the first-stage purchase threshold is obtained by substituting $p_{1}^{DL*}$ into the indifference equation $f_{1}^{DL}\left( Q \right)\cdot\left( v-p_{1}^{DL} \right)=\delta\sum_{x_{1}=0}^{Q} \left( f_{2}^{DL}\left( q \right)\cdot\left( v-\hat{p}_{2}^{DL}\left( q \right) \right) \right)\cdot Pr(D_{1}^{DL}=x_{1})$. Since $\hat{p}_{2}^{DL}\left( q \right)$ is formed by Rational Expectation Equilibrium, there is $\hat{p}_{2}^{DL}\left( q \right)=p_{2}^{DL*}$. Therefore, the optimal first-stage purchase threshold ${v=v}_{1}^{DL*}\left( p_{1}^{DL*} \right)$ is the solution to $f_{1}^{DL}\left( Q \right)\cdot\left( v-p_{1}^{DL*} \right)=\delta\sum_{x_{1}=0}^{Q} \left( f_{2}^{DL}\left( q \right)\cdot\left( v-p_{2}^{DL*} \right) \right)\cdot Pr(D_{1}^{DL}=x_{1})$.

Given the optimal first-stage price $p_{1}^{DL*}$*,* the equilibrium indifference equation$f_{1}^{DL}\left( Q \right)\cdot\left( v_{1}^{DL*}\left( p_{1}^{DL*} \right)-p_{1}^{DL*} \right)=\delta\sum_{x_{1}=0}^{Q} \left( f_{2}^{DL}\left( q \right)\cdot\left( v_{1}^{DL*}\left( p_{1}^{DL*} \right)-p_{2}^{DL*} \right) \right)\cdot Pr(D_{1}^{DL}=x_{1})$ can be rewritten as:

$$\begin{aligned} v_{1}^{DL*}\left( p_{1}^{DL*} \right)=\frac{\delta\sum_{x_{1}=0}^{Q} f_{2}^{K}\left( q \right)\cdot\left( v_{1}^{DL*}\left( p_{1}^{DL*} \right)-p_{2}^{DL*} \right)\cdot\Pr\left( D_{1}^{K}=x_{1} \right)}{f_{1}^{K}\left( Q \right)}+p_{1}^{DL*}\#\left( A8 \right) \end{aligned}$$

In fact, Eq. ([A8](#A8)) is a fixed-point problem, Note that the right-hand side of Eq. ([A8](#A8)) is increasing in $\delta$, therefore, the solution to Eq. ([A8](#A8)) is increasing in $\delta$.

Given the optimal first-stage purchase threshold $v_{1}^{DL*}\left( p_{1}^{DL*} \right)$, Let the conditional distribution in the second-stage demand be $\bar{D_{2}^{DL}}\sim Poisson\left[ \left( v_{1}^{DL*}\left( p_{1}^{DL*} \right)-p_{2}^{DL} \right)\tilde{\lambda}|\tilde{\lambda} \right]$. Then the seller's second-stage expected revenue $R_{2}^{DL}=p_{2}^{DL}\cdot E_{D_{2}^{DL}}\left[ \min\left( D_{2}^{DL},q \right) \right]$ in the text can be rewritten as:

$$\begin{aligned} R_{2}^{DL}=\int_{0}^{\infty} \left\{ {p_{2}^{DL}\cdot E}_{\bar{D_{2}^{DL}}}\left[ \min\left( q,\bar{D_{2}^{DL}} \right) \right] \right\}f\left( \tilde{\lambda} \right)d\tilde{\lambda}\#\left( A9 \right) \end{aligned}$$

And the first-order and second-order derivatives of the Eq. ([A9](#A9)) with respect to $p_{2}^{DL}$ are as follows:

$$\begin{aligned} \frac{\partial R_{2}^{DL}}{\partial p_{2}^{DL}}=\int_{0}^{\infty} \left\{ E_{\bar{D_{2}}}\left[ \min\left( q,\bar{D_{2}^{DL}} \right) \right]-p_{2}^{DL}\tilde{\lambda}\sum_{x=0}^{q-1} P\left( \bar{D_{2}^{DL}}=x \right) \right\}f\left( \tilde{\lambda} \right)d\tilde{\lambda}\#\left( A10 \right) \end{aligned}$$

$$\begin{aligned} \frac{\partial^{2}R_{2}^{DL}}{\partial\left( p_{2}^{DL} \right)^{2}}=-\int_{0}^{\infty} \left\{ 2\tilde{\lambda}\sum_{x=0}^{q-1} P\left( \bar{D_{2}^{DL}}=x \right)+p_{2}^{DL}\tilde{\lambda}^{2}P(\bar{D_{2}^{DL}}=q-1) \right\}f\left( \tilde{\lambda} \right)d\tilde{\lambda}<0\#\left( A11 \right) \end{aligned}$$

where the expression of $f(\tilde{\lambda})$ is similar to Eq. ([A2](#A2)), but parameters are $(\alpha+x_{1},\beta+1-v_{1}^{DL*}\left( p_{1}^{DL*} \right))$.

Since Eq. ([A11](#A11)) is less than zero, the expected revenue of the second stage is proved to be a strictly concave function of $p_{2}^{DL}$. When $p_{2}^{DL}=v_{1}^{DL*}\left( p_{1}^{DL*} \right)$, the second stage price is so high that no consumers buy the product, and there is $R_{2}^{DL}|_{p_{2}^{DL}=v_{1}^{DL*}\left( p_{1}^{DL*} \right)}=R_{2}^{DL}|_{p_{2}^{DL}=0}=0$, thus the optimal price $p_{2}^{DL*}\in[0,v_{1}^{DL*}\left( p_{1}^{DL*} \right)]$ that maximizes the expected revenue.

Theorem 2 is proved.

**Proof of Theorem 3**

Given the optimal first-stage price $p_{1}^{GL*}$ and any sales data $x_{1}$, let the conditional distribution in the second-stage demand be $\bar{D_{2}^{GL}}\sim Poisson\left[ \left( p_{1}^{GL*}-p_{2}^{GL} \right)\tilde{\lambda}|\tilde{\lambda} \right]$ under strategy $GL$, thus the second-stage expected revenue function $R_{2}^{GL}=p_{2}^{GL}\cdot E_{D_{2}^{GL}}\left[ \min\left( D_{2}^{GL},q \right) \right]-x_{1}\left( p_{1}^{GL*}-p_{2}^{GL} \right)^{+}$ in our text is substituted as:

$$\begin{aligned} R_{2}^{GL}=\int_{0}^{\infty} \left\{ {p_{2}^{GL}\cdot E}_{\bar{D_{2}^{GL}}}\left[ \min\left( q,\bar{D_{2}^{GL}} \right) \right]-x_{1}\left( p_{1}^{GL*}-p_{2}^{GL} \right)^{+} \right\}f\left( \tilde{\lambda} \right)d\tilde{\lambda}\#\left( A12 \right) \end{aligned}$$

The first-order and second-order derivatives of the Eq. ([A12](#A12)) with respect to $p_{2}^{GL}$ are as follows:

$$\begin{aligned} \frac{\partial R_{2}^{GL}}{\partial p_{2}^{GL}}=\int_{0}^{\infty} \left\{ E_{\bar{D_{2}^{GL}}}\left[ \min\left( q,\bar{D_{2}^{GL}} \right) \right]-p_{2}^{GL}\tilde{\lambda}\sum_{x=0}^{q-1} P\left( \bar{D_{2}^{GL}}=x \right) \right\}f\left( \tilde{\lambda} \right)d\tilde{\lambda}+A\#\left( A13 \right) \end{aligned}$$

$$\begin{aligned} \frac{\partial^{2}R_{2}^{GL}}{\partial\left( p_{2}^{GL} \right)^{2}}=-\int_{0}^{\infty} \left\{ 2\tilde{\lambda}\sum_{x=0}^{q-1} P\left( \bar{D_{2}^{GL}}=x \right)+p_{2}^{GL}\tilde{\lambda}^{2}P\left( \bar{D_{2}^{GL}}=q-1 \right) \right\}f\left( \tilde{\lambda} \right)d\tilde{\lambda}<0\#\left( A14 \right) \end{aligned}$$

where $A=\left\{ \begin{aligned} &0,p_{2}^{GL}\geq p_{1}^{GL*} \\ &x_{1},p_{2}^{GL}<p_{1}^{GL*} \end{aligned} \right.$, is independent of $p_{2}^{GL}$, and $f\left( \tilde{\lambda} \right)$ is the Bayesian updated Gamma distribution with parameters $(\alpha+x_{1},\beta+1-p_{1}^{GL*})$.

According to Eq. ([A14](#A14)), the second-order derivative of the expected revenue in the second stage is strictly negative, so it is a strictly concave function with respect to $p_{2}^{GL}$. There is no positive revenue in the second stage because of higher price $p_{2}^{GL}=p_{1}^{GL*}$, that is, $R_{2}^{GL}|_{p_{2}^{GL}=p_{1}^{GL*}}=R_{2}^{GL}|_{p_{2}^{GL}=0}=0$, so $p_{2}^{GL*}$ ranges in the interval of $[0,p_{1}^{GL*}]$.

As proved in Theorem 1 and Proposition 1, for price matching with demand learning, consumers purchase a product in the first stage as long as $v\geq p_{1}^{GL}$. Therefore, two-stage estimated demand distribution is $D_{1}^{GL}\sim NB\left( \alpha,\frac{\beta}{\beta+1-p_{1}^{GL}} \right)$and $D_{2}^{GL}\sim NB\left( \alpha+x_{1},\frac{\beta+1-p_{1}^{GL}}{\beta+1-p_{2}^{GL}} \right)$, respectively. Since the demand in two stages is independent of $\delta$, the two-stage price and total expected revenue have nothing to do with $\delta$.

Theorem 3 is proved.

**Appendix B：Numerical Results**

The following three tables summarize the results of strategies $K=\{DN,DL,GN,GL\}$ in the actual market size $\lambda=\{10,10,30\}$, respectively. Note that (i) the first term in parentheses is optimal inventory $Q^{*}$, and the second term represents the profit of the corresponding model; (ii) the symbol "-" in the table indicates that the profit is negative; (iii) the underlined term reflects the profit of the optimal strategy.

**Table B1.** The results of strategy $K$ when $\lambda=10$

| $c$ | $CV$ | Model or $b^{DP}$/$b^{GP}$ | $\delta$ | | | |
| --- | --- | --- | --- | --- | --- | --- |
|  |  |  | 0.2 | 0.4 | 0.6 | 0.8 |
| 0.1 | 0.1118 | $DL$ | (10,0.6010) | (9,0.6053) | (9,0.5468) | (8,0.4603) |
|  |  | $DN$ | (10,0.6009) | (9,0.5575) | (9,0.4504) | (8,0.4575) |
|  |  | $\boldsymbol{b}^{\boldsymbol{DP}}$ | 0.01% | 2.24% | 4.77% | 0.15% |
|  |  | $GL$ | (7,1.1464) | (7,1.1464) | (7,1.1464) | (7,1.1464) |
|  |  | $GN$ | (7,1.1463) | (7,1.1463) | (7,1.1463) | (7,1.1463) |
|  |  | $\boldsymbol{b}^{\boldsymbol{GP}}$ | 0.01% | 0.01% | 0.01% | 0.01% |
|  | 0.2236 | $DL$ | (10,0.5973) | (9,0.5511) | (9,0.4404) | (8,0.3945) |
|  |  | $DN$ | (10,0.5986) | (9,0.5547) | (9,0.4936) | (8,0.5023) |
|  |  | $\boldsymbol{b}^{\boldsymbol{DP}}$ | -0.06% | -0.17% | -2.63% | -5.64% |
|  |  | $GL$ | (7,1.0788) | (7,1.0788) | (7,1.0788) | (7,1.0788) |
|  |  | $GN$ | (7,1.0786) | (7,1.0786) | (7,1.0786) | (7,1.0786) |
|  |  | $\boldsymbol{b}^{\boldsymbol{GP}}$ | 0.01% | 0.01% | 0.01% | 0.01% |
|  | 1 | $DL$ | (12,-) | (12,-) | (11,-) | (10,-) |
|  |  | $DN$ | (12,0.1372) | (11,0.1086) | (10,-) | (9,-) |
|  |  | $GL$ | (9,0.5710) | (9,0.5710) | (9,0.5710) | (9,0.5710) |
|  |  | $GN$ | (9,0.3711) | (9,0.3711) | (9,0.3711) | (9,0.3711) |
|  |  | $\boldsymbol{b}^{\boldsymbol{GP}}$ | 10.64% | 10.64% | 10.64% | 10.64% |
| 0.2 | 0.1118 | $DL$ | (7,-) | (6,-) | (6,-) | (5,-) |
|  |  | $DN$ | (7,-) | (6,-) | (6,-) | (5,-) |
|  |  | $GL$ | (5,0.3140) | (5,0.3140) | (5,0.3140) | (5,0.3140) |
|  |  | $GN$ | (5,0.3138) | (5,0.3138) | (5,0.3138) | (5,0.3138) |
|  |  | $\boldsymbol{b}^{\boldsymbol{GP}}$ | 0.01% | 0.01% | 0.01% | 0.01% |
|  | 0.2236 | $DL$ | (7,-) | (6,-) | (6,-) | (5,-) |
|  |  | $DN$ | (7,-) | (6,-) | (6,-) | (5,-) |
|  |  | $GL$ | (5,0.2745) | (5,0.2745) | (5,0.2745) | (5,0.2745) |
|  |  | $GN$ | (5,0.2724) | (5,0.2724) | (5,0.2724) | (5,0.2724) |
|  |  | $\boldsymbol{b}^{\boldsymbol{GP}}$ | 0.15% | 0.15% | 0.15% | 0.15% |
|  | 1 | $DL$ | (6,-) | (6,-) | (5,-) | (5,-) |
|  |  | $DN$ | (7,-) | (6,-) | (6,-) | (5,-) |
|  |  | $GL$ | (4,-) | (4,-) | (4,-) | (4,-) |
|  |  | $GN$ | (5,-) | (5,-) | (5,-) | (5,-) |

**Table B2.** The results of strategy $K$ when $\lambda=20$

| $c$ | $CV$ | Model or $b^{DP}$/$b^{GP}$ | $\delta$ | | | |
| --- | --- | --- | --- | --- | --- | --- |
|  |  |  | 0.2 | 0.4 | 0.6 | 0.8 |
| 0.1 | 0.1118 | $DL$ | (12,2.9743) | (12,2.8775) | (12,2.6585) | (12,2.4897) |
|  |  | $DN$ | (12,2.9703) | (12,2.7999) | (12,2.5059) | (12,2.4767) |
|  |  | $\boldsymbol{b}^{\boldsymbol{DP}}$ | 0.08% | 1.72% | 3.56% | 0.32% |
|  |  | $GL$ | (10,3.418) | (10,3.418) | (10,3.418) | (10,3.418) |
|  |  | $GN$ | (10,3.4207) | (10,3.4207) | (10,3.4207) | (10,3.4207) |
|  |  | $\boldsymbol{b}^{\boldsymbol{GP}}$ | -0.07% | -0.07% | -0.07% | -0.07% |
|  | 0.2236 | $DL$ | (12,2.9404) | (11,2.7885) | (11,2.4871) | (11,2.3827) |
|  |  | $DN$ | (12,2.9658) | (11,2.7964) | (11,2.5818) | (11,2.5375) |
|  |  | $\boldsymbol{b}^{\boldsymbol{DP}}$ | -0.54% | -0.18% | -2.21% | -3.78% |
|  |  | $GL$ | (10,3.3165) | (10,3.3165) | (10,3.3165) | (10,3.3165) |
|  |  | $GN$ | (10,3.3225) | (10,3.3225) | (10,3.3225) | (10,3.3225) |
|  |  | $\boldsymbol{b}^{\boldsymbol{GP}}$ | -0.16% | -0.16% | -0.16% | -0.16% |
|  | 1 | $DL$ | (15,1.7116) | (15,1.2702) | (14,0.7820) | (13,0.2124) |
|  |  | $DN$ | (15,2.0296) | (14,1.8789) | (13,1.5706) | (12,1.3848) |
|  |  | $\boldsymbol{b}^{\boldsymbol{DP}}$ | -6.72% | -13.5% | -18.38% | -28.66% |
|  |  | $GL$ | (12,2.2002) | (12,2.2002) | (12,2.2002) | (12,2.2002) |
|  |  | $GN$ | (12,2.5532) | (12,2.5532) | (12,2.5532) | (12,2.5532) |
|  |  | $\boldsymbol{b}^{\boldsymbol{GP}}$ | -9.13% | -9.13% | -9.13% | -9.13% |
| 0.2 | 0.1118 | $DL$ | (9,2.2940) | (8,2.2578) | (8,2.0265) | (7,2.1304) |
|  |  | $DN$ | (9,2.2336) | (8,2.1820) | (8,2.0228) | (7,2.0622) |
|  |  | $\boldsymbol{b}^{\boldsymbol{DP}}$ | 1.71% | 2.27% | 0.12% | 2.25% |
|  |  | $GL$ | (7,2.4621) | (7,2.4621) | (7,2.4621) | (7,2.4621) |
|  |  | $GN$ | (7,2.4628) | (7,2.4628) | (7,2.4628) | (7,2.4628) |
|  |  | $\boldsymbol{b}^{\boldsymbol{GP}}$ | -0.02% | -0.02% | -0-2.40%.02% | -0.02% |
|  | 0.2236 | $DL$ | (9,2.2114) | (8,2.1669) | (8,2.0036) | (7,1.9859) |
|  |  | $DN$ | (9,2.2324) | (8,2.2401) | (8,2.1306) | (7,2.0586) |
|  |  | $\boldsymbol{b}^{\boldsymbol{DP}}$ | -0.60% | -2.19% | -3.98% | -2.40% |
|  |  | $GL$ | (7,2.4089) | (7,2.4089) | (7,2.4089) | (7,2.4089) |
|  |  | $GN$ | (7,2.4193) | (7,2.4193) | (7,2.4193) | (7,2.4193) |
|  |  | $\boldsymbol{b}^{\boldsymbol{GP}}$ | -0.36% | -0.36% | -0.36% | -0.36% |
|  | 1 | $DL$ | (8,1.7221 | (8,1.5511) | (7,1.2323) | (7,0.9764) |
|  |  | $DN$ | (9,1.8501) | (8,1.6767) | (8,1.5297) | (7,1.7015 |
|  |  | $\boldsymbol{b}^{\boldsymbol{DP}}$ | -3.63% | -3.75% | -9.33% | -23.91% |
|  |  | $GL$ | (6,1.7817) | (6,1.7817) | (6,1.7817) | (6,1.7817) |
|  |  | $GN$ | (7,1.9485) | (7,1.9485) | (7,1.9485) | (7,1.9485) |
|  |  | $\boldsymbol{b}^{\boldsymbol{GP}}$ | -5.79% | -5.79% | -5.79% | -5.79% |

**Table B3.** The results of strategy $K$ when $\lambda=30$

| $c$ | $CV$ | Model or $b^{DP}$/$b^{GP}$ | $\delta$ | | | |
| --- | --- | --- | --- | --- | --- | --- |
|  |  |  | 0.2 | 0.4 | 0.6 | 0.8 |
| 0.1 | 0.1118 | $DL$ | (14,5.7739) | (13,5.2960) | (13,4.8816) | (11,4.5143) |
|  |  | $DN$ | (14,5.7792) | (13,5.3214) | (13,4.9206) | (11,4.5298) |
|  |  | $\boldsymbol{b}^{\boldsymbol{DP}}$ | -0.07% | -0.37% | -0.59% | -0.25% |
|  |  | $GL$ | (11,5.1556) | (11,5.1556) | (11,5.1556) | (11,5.1556) |
|  |  | $GN$ | (11,5.1504) | (11,5.1504) | (11,5.1504) | (11,5.1504) |
|  |  | $\boldsymbol{b}^{\boldsymbol{GP}}$ | 0.09% | 0.09% | 0.09% | 0.09% |
|  | 0.2236 | $DL$ | (14,5.7415) | (13,5.2885) | (13,4.8711) | (12,4.4978) |
|  |  | $DN$ | (14,5.7802) | (13,5.3121) | (13,4.9230) | (12,4.5485) |
|  |  | $\boldsymbol{b}^{\boldsymbol{DP}}$ | -0.53% | -0.34% | -0.78% | -0.80% |
|  |  | $GL$ | (11,5.1507) | (11,5.1507) | (11,5.1507) | (11,5.1507) |
|  |  | $GN$ | (11,5.1388) | (11,5.1388) | (11,5.1388) | (11,5.1388) |
|  |  | $\boldsymbol{b}^{\boldsymbol{GP}}$ | 0.20% | 0.20% | 0.20% | 0.20% |
|  | 1 | $DL$ | (17,4.4051) | (17,3.7224) | (16,3.0707) | (15,2.3714) |
|  |  | $DN$ | (17,4.8982) | (16,4.6646) | (15,4.2645) | (14,3.9794) |
|  |  | $\boldsymbol{b}^{\boldsymbol{DP}}$ | -6.80% | -13.61% | -18.05% | -25.44% |
|  |  | $GL$ | (14,4.5668) | (14,4.5668) | (14,4.5668) | (14,4.5668) |
|  |  | $GN$ | (14,4.3380) | (14,4.3380) | (14,4.3380) | (14,4.3380) |
|  |  | $\boldsymbol{b}^{\boldsymbol{GP}}$ | 3.93% | 3.93% | 3.93% | 3.93% |
| 0.2 | 0.1118 | $DL$ | (11,4.3613) | (10,3.9783) | (10,3.7060) | (9,3.4015) |
|  |  | $DN$ | (11,4.3686) | (10,3.9849) | (10,3.7093) | (9,3.4093) |
|  |  | $\boldsymbol{b}^{\boldsymbol{DP}}$ | -0.13% | -0.13% | -0.07% | -0.16% |
|  |  | $GL$ | (9,3.8356) | (9,3.8356) | (9,3.8356) | (9,3.8356) |
|  |  | $GN$ | (9,3.8355) | (9,3.8355) | (9,3.8355) | (9,3.8355) |
|  |  | $\boldsymbol{b}^{\boldsymbol{GP}}$ | 0.00% | 0.00% | 0.00% | 0.00% |
|  | 0.2236 | $DL$ | (11,4.3581) | (10,3.9627) | (10,3.6873) | (9,3.3824) |
|  |  | $DN$ | (11,4.3635) | (10,3.9831) | (10,3.7109) | (9,3.4038) |
|  |  | $\boldsymbol{b}^{\boldsymbol{DP}}$ | -0.10% | -0.39% | -0.47% | -0.45% |
|  |  | $GL$ | (9,3.8448) | (9,3.8448) | (9,3.8448) | (9,3.8448) |
|  |  | $GN$ | (9,3.8380) | (9,3.8380) | (9,3.8380) | (9,3.8380) |
|  |  | $\boldsymbol{b}^{\boldsymbol{GP}}$ | 0.15% | 0.15% | 0.15% | 0.15% |
|  | 1 | $DL$ | (10,4.0014) | (10,3.6493) | (9,3.1591) | (9,2.8852) |
|  |  | $DN$ | (11,4.1992) | (10,3.8566) | (10,3.6191) | (9,3.3543) |
|  |  | $\boldsymbol{b}^{\boldsymbol{DP}}$ | -8.62% | -3.98% | -9.24% | -9.86% |
|  |  | $GL$ | (8,3.4787) | (8,3.4787) | (8,3.4787) | (8,3.4787) |
|  |  | $GN$ | (9,3.4557) | (9,3.4557) | (9,3.4557) | (9,3.4557) |
|  |  | $\boldsymbol{b}^{\boldsymbol{GP}}$ | 0.52% | 0.52% | 0.52% | 0.52% |
