# Supplementary figures and images for: Dynamic pricing strategies towards strategic consumers under demand learning
Source: PLoS One. 2026 Jan 2;21(1):e0340105. doi: 10.1371/journal.pone.0340105 (PMC12758830; doi:10.1371/journal.pone.0340105)

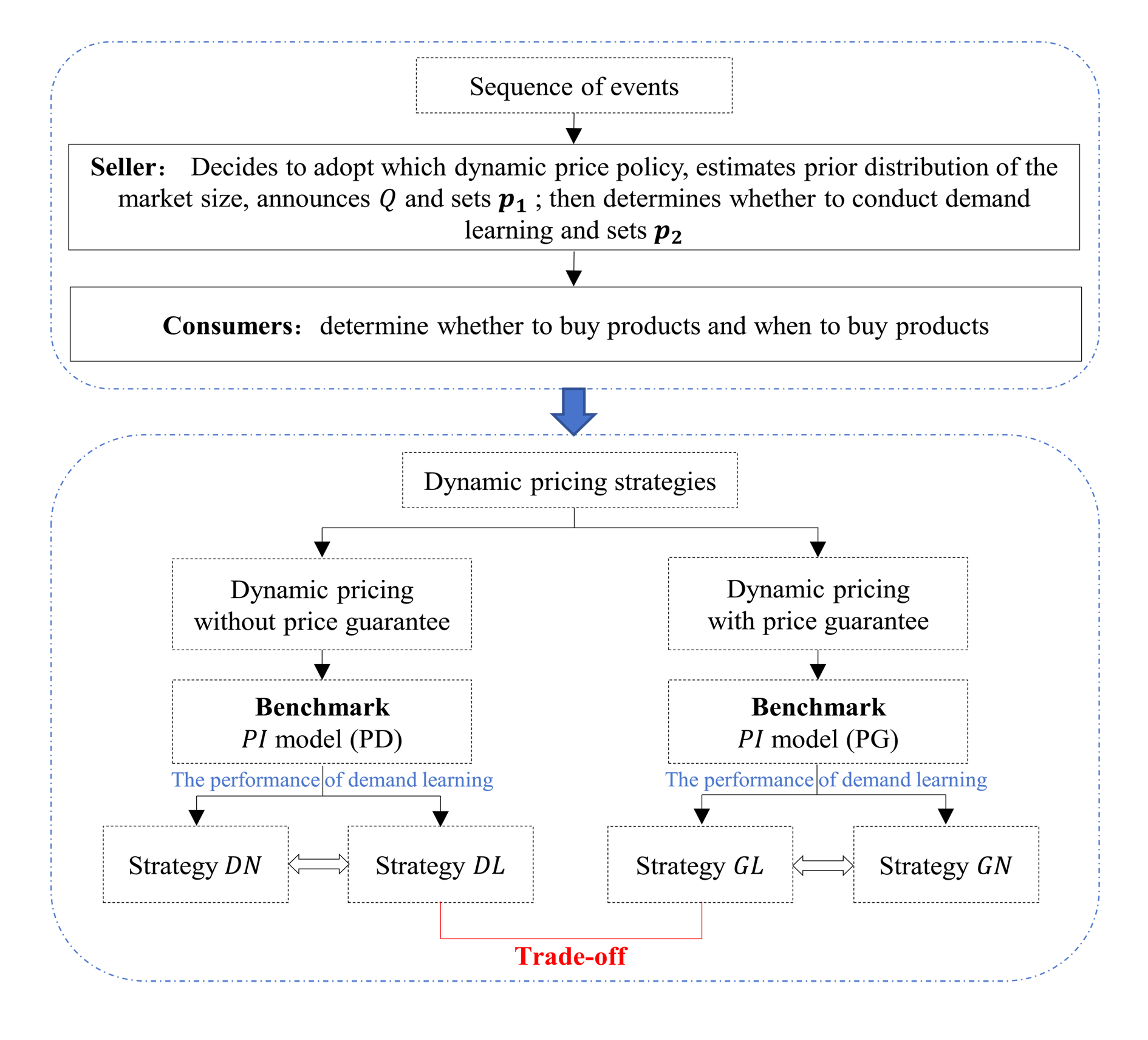

Supplement: S1 Fig — (TIF) [file pone.0340105.s002.tif]

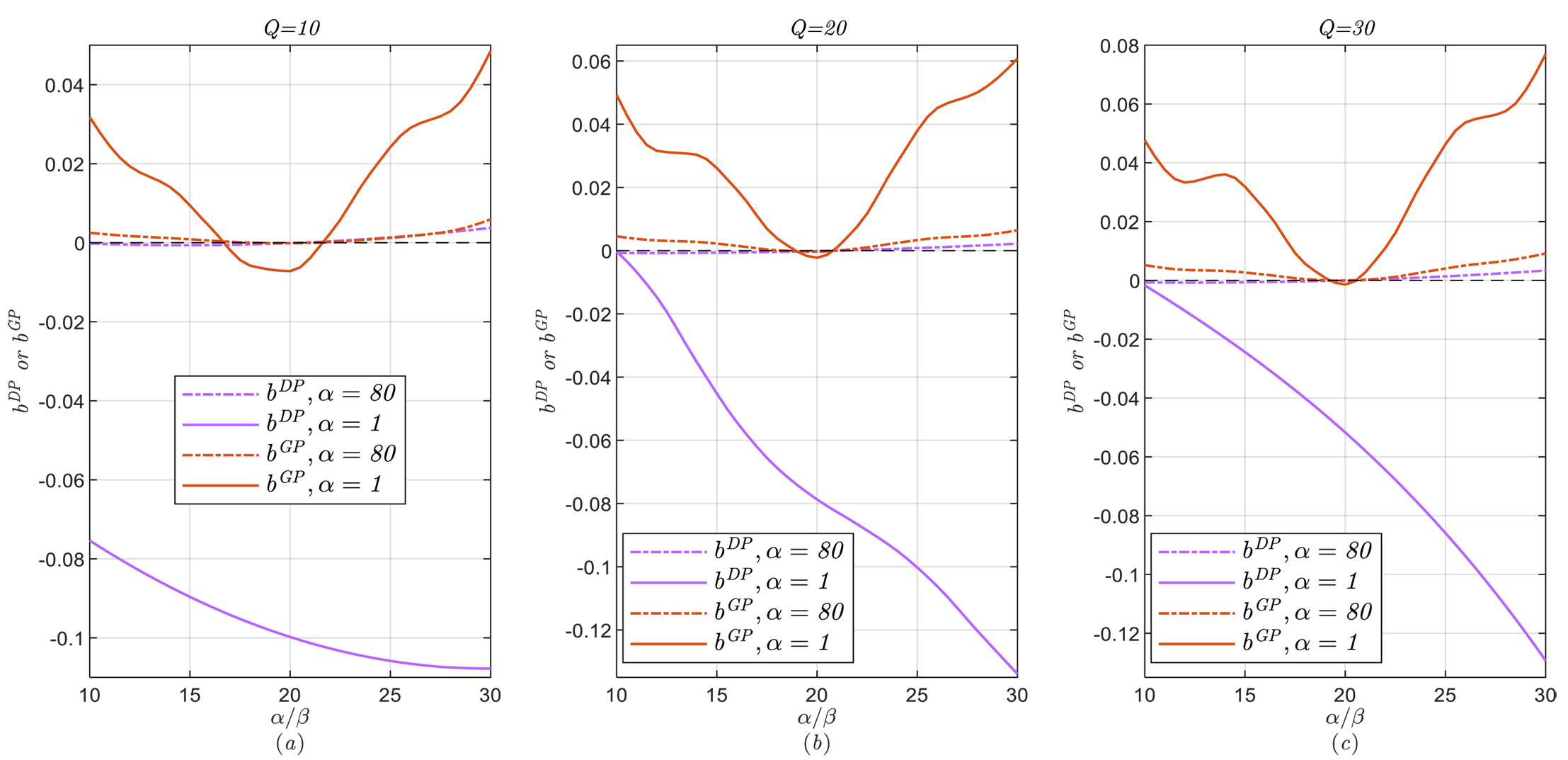

Supplement: S2 Fig — (TIF) [file pone.0340105.s003.tif]

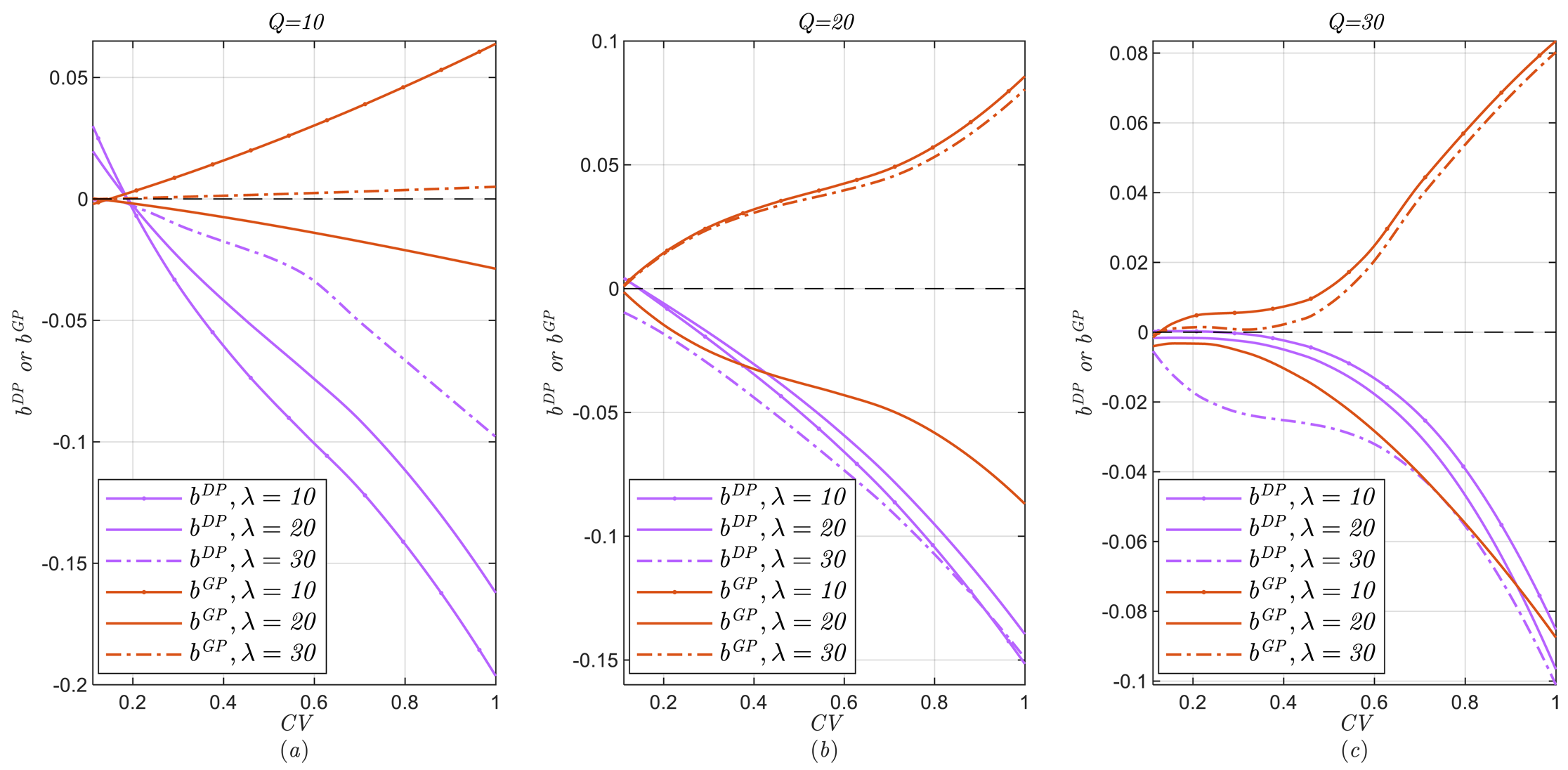

Supplement: S3 Fig — (TIF) [file pone.0340105.s004.tif]
