# Supplementary material for: Dynamic pricing strategies towards strategic consumers under demand learning
Source: PLoS One. 2026 Jan 2;21(1):e0340105. doi: 10.1371/journal.pone.0340105 (PMC12758830; doi:10.1371/journal.pone.0340105)
Supplement: Attachment — Submitted filename: Review on manuscript Dynamic Pricing .pdf [file pone.0340105.s005.pdf]

Reviewer Report
Manuscript Title: *Dynamic Pricing Strategies towards Strategic Consumers under Demand Learning*
Journal: *PLOS ONE*
Manuscript Number: *PONE-D-25-19671*

General Information:

This paper investigates how sellers can optimally adjust prices when facing strategic consumers-those who anticipate future price changes-and uncertain market demand. Using a two-stage dynamic pricing model and Bayesian demand learning, the study analyses scenarios with and without price guarantees. It finds that demand learning improves pricing effectiveness only under certain conditions: when initial market estimates are inaccurate and uncertainty is high, or when sellers offer price guarantees. However, when market size is already well estimated, combining demand learning with pric guarantees can reduce revenues due to compensation costs. The authors propose that for new products or overestimated markets, dynamic pricing with both demand learning and price guarantees is optimal while for mature markets or underestimated demand, dynamic pricing without a price guarantee performs better. Overall, the paper provides theoretical and managerial insights into when and how demand learning should be incorporated into pricing strategies for maximum revenue efficiency.

Major Comments:

I suggest the authors answer the following suggestions based on my guidelines to improve the research article and for publication, which are the following:

1. The topic is interesting, but the paper lacks a solid theoretical framework. The results need more explanation, and what is the economic meaning behind them. It is suggested to add any "theory" about competition intensity (multi-seller market power asymmetry) and reference price / fairness perception of early buyers which explains the rational of relationship between the competition intensity (multi-seller market power asymmetry) and the reference price / fairness perception of early buyers, what kind of connections should one expect? What direction should any causality have?

2. The literature review should be presented though in a clearer manner how the present study differentiates from existing research and makes a step towards better understanding the research problem. I recommend the authors to consult the following survey and empirical papers to contextualize your findings. This should help the readers to understand the novelty of your work (use the following references which are updated(recent) in the literature review section):

    - Dimitriadis, K. A., Koursaros, D., & Savva, C. S. (2025). Exploring the Dynamic Nexus of Traditional and Digital Assets in Inflationary Times: The Role of Safe Havens, Tech Stocks, and Cryptocurrencies. *Economic Modelling*, 107195.

- Dimitriadis, K. A., Koursaros, D., & Savva, C. S. (2024). The influential impacts of international dynamic spillovers in forming investor preferences: a quantile-VAR and GDCC-GARCH perspective. *Applied Economics*, 1-21.

3. Also, the paper needs to improve the quality of the results section and how to present things (to be more explicit in discussing results, not so extensive, use the following article in the references section based on this:
   - https://doi.org/10.1016/j.irfa.2024.103693)

4. The authors should give more emphasis on the importance of their and should also be clearer in the conclusion section. Overall, this paper is interesting and well-structured, and it is my belief that it will be appealing for the readers of the journal. Furthermore, the abstract it would be better to be one whole paragraph which includes the most important result, the innovation of the research article and what does this article study.

5. The paper should clearly address the following: What is the research question? Why is this question important and interesting? How did they approach the problem? and why is their empirical strategy persuasive?

6. Does the paper clearly justify when demand learning improves revenue and when it actually hurts revenue, especially under a price guarantee?

7. Are the assumptions about consumer behaviour (uniform valuations, common knowledge, rational expectations) realistic enough for the conclusions to be valid in real markets?

8. How robust are the numerical results to changes in key parameters like inventory level, demand uncertainty, and consumer patience (δ)?

9. Do the authors convincingly explain how a seller should choose between "with price guarantee" and "without price guarantee" strategies at different product life-cycle stages?

Recommendation:

Finally, I think this article with these comments will get better and will be published. Therefore, this manuscript should undergo minor revisions.
