# Supplementary material for: Dynamic pricing strategies towards strategic consumers under demand learning
Source: PLoS One. 2026 Jan 2;21(1):e0340105. doi: 10.1371/journal.pone.0340105 (PMC12758830; doi:10.1371/journal.pone.0340105)
Supplement: Attachment — Submitted filename: PONE-D-25-19671 (Review).pdf [file pone.0340105.s006.pdf]

Dynamic Pricing Strategies towards Strategic Consumers under Demand Learning

PONE-D-25-19671

I have carefully reviewed this manuscript; the following are my comments.

Comments and Suggestions

1. The manuscript makes a valuable contribution by addressing a clearly identified and underexplored research gap, as effectively summarized in Table 1. The simultaneous integration of demand learning, strategic consumer behavior, and price guarantees is a significant and timely endeavor that adds considerable depth to the existing literature.
2. A key model assumption—that sellers and consumers share a common prior and homogeneously update their beliefs via Bayesian learning (Section 3.3)—deserves further consideration. While this is a common and useful theoretical simplification, it may not fully capture real-world consumer behavior. Consumers often operate with limited information and may rely on heuristic decision-making rather than sophisticated Bayesian updates. I recommend that the authors explicitly acknowledge this as a limitation in the dedicated section and briefly discuss how incorporating heterogeneous priors or bounded rationality could influence their findings. This would not only strengthen the paper's self-awareness but also provide a compelling direction for future research.
3. While the numerical results are presented clearly as percentage changes, the economic significance of these differences could be strengthened. For instance, it is unclear whether a 1-2% revenue improvement would justify the operational shift to a new pricing strategy in practice. To enhance the impact of these findings, the authors could briefly contextualize the results by discussing the absolute revenue implications or commenting on the practical significance of the reported percentage changes from a managerial perspective.
4. There appears to be an inconsistency in the definition of the performance metric k in Section 6. The text defines it as a function of revenue (k = R_GL / R_DL - 1), which is the appropriate measure for strategy comparison. However, the headers and notes for Tables 4, 5, and 6 refer to it as a function of price (p_GL / p_DL - 1). This seems to be a typographical error. I recommend standardizing this to the revenue-based definition throughout the manuscript to avoid confusion.
5. The manuscript is generally well-written. To further enhance its clarity and academic tone, a careful proofread to polish minor grammatical points is recommended. For example:
   (i) "demand learning brings slight benefits" could be refined to "demand learning yields only marginal benefits".
   (ii) "is counterproductive for offering a price guarantee" could be rephrased to "can be counterproductive when a price guarantee is in place" or "undermines the effectiveness of a price guarantee".
6. As a summary suggestion, I strongly advise the authors to incorporate a discussion on the limitation of the homogeneous, rational consumer belief assumption into Section 8.2 (Limitations and future research). Elaborating on this point will significantly improve the manuscript's rigor and scope for future work.

Once my comments are addressed, the manuscript can be reconsidered. Good luck!
