# Supplementary material for: Dynamic pricing strategies towards strategic consumers under demand learning
Source: PLoS One. 2026 Jan 2;21(1):e0340105. doi: 10.1371/journal.pone.0340105 (PMC12758830; doi:10.1371/journal.pone.0340105)
Supplement: Attachment — Submitted filename: plos one-Response to Reviewers.docx [file pone.0340105.s008.docx]

**Authors’ response to reviewers’ for “****Dynamic Pricing Strategies towards Strategic Consumers under Demand Learning”**

Dear Editors and Reviewers:

We are deeply grateful for the opportunity to revise our manuscript entitled “**Dynamic Pricing Strategies towards Strategic Consumers under Demand Learning**” (ID: **PONE-D-25-19671**)

We sincerely thank the editors and all reviewers for their insightful comments, which have been invaluable in enhancing the quality of our manuscript. We have studied these suggestions carefully and made point-by-point corrections and improvements which are **marked in red** in the **revised manuscript**. We hope that these revisions meet with your approval.

Yours sincerely,

The authors

**A point-by-point response to the reviewers’ comments**

**Review comments from reviewer 1**

**Major concerns:**

1. ***The topic is interesting, but the paper lacks a solid theoretical framework. The results need more explanation, and what is the economic meaning behind them. It is suggested to add any “theory” about competition intensity (multi-seller market power asymmetry) and reference price / fairness perception of early buyers which explains the rational of relationship between the competition intensity (multi-seller market power asymmetry) and the reference price / fairness perception of early buyers, what kind of connections should one expect? What direction should any causality have?***

**Our response:** We greatly appreciate your comments. We employ rational expectation equilibrium (REE) introduced by Muth (1961) as the theoretical framework to study dynamic pricing strategies for a seller facing strategic consumers. REE believes that if decision-makers are completely rational and information is fully utilized, their expectations of future outcomes are consistent with the actual results. Since Su (2007) first used REE to describe the interaction between strategic consumers’ purchasing strategy and a seller’s decisions, later massive mainstream literature on strategic consumers also adopt the theory, such as Su and Zhang (2007), Cachon and Swinney (2008), Shum et al. (2016) and Cao et al. (2022). Following them, in our paper does not consider reference price effect/ fairness perception. REE emphasizes that when consumers first enter the market, they form correct beliefs about the seller’s second-stage price and other consumer behaviors, obtaining two-stage expected surplus to make purchasing decisions. And the seller also correctly anticipates consumers' purchasing behavior, obtains a two-stage demand distribution, and determines two-stage optimal prices. The details and modifications are the red text in **paragraph 4 of Section 3.1 on pages 9-10**. **Other relevant revisions can be found in paragraphs 1-4 and Table 3 of Section 3.4 on pages 13-14**, **paragraph 1 of Section 4.1 on page 16**, **paragraphs 1-2 of Section 4.2 on page 17**, **paragraph 1 of Section 4.3 on page 18**, **and paragraphs 1-2 of Section 4.4 on pages 18-19**.

In addition, thank you very much for your suggestion to consider competition intensity. However, when demand is uncertain, a seller cannot accurately know the parameters of its own function and the parameters of its competitors’ functions. it is impossible to describe the correlation of demand between him and competitors (Chen and Chen, 2015; Famil and Seif, 2024)). Dynamic pricing under competitive conditions has received little attention until recently. In fact, solving a dynamic-pricing problem under competitive conditions, demand modeling, and price optimization are generally more complex than problems without competition (Famil and Seif, 2024).

More importantly, we believe that our models are reasonable. Our explanations are as follows.

(1) Dynamic pricing with demand learning is more achievable for a seller than multiple sellers. In the sales process, the seller knows his own historical prices and sales data, but it is difficult to know the behavior of competitors. Even if the seller knows the historical prices of competitors, he cannot obtain competitors’ true historical sales data (Chen and Chen, 2015; den Boer, 2015). Therefore, it is not easy to implement demand learning in competitive environments.

(2) The monopoly model and competition outcomes may be consistent. Chen and Chen (2015) and Cooper et al. (2015) state that monopoly models include competition effects because the sales data used for demand learning comes from real competitive markets. Cooper et al. (2015) refer to this theory as the market response hypothesis and validate its effectiveness by studying the duopoly model. They find that this hypothesis is valid under certain conditions.

(3) Our results are also consistent with the empirical strategies of competing sellers in business practices. Taking JD.com as an example, the results we obtain based on numerical results are the same as the strategies of JD.com. However, our results are also aligned with Taobao's strategies, who is a strong competitor of JD.com.

Based on the above mentioned, we do not consider competition in our paper. Nevertheless, in the revised version, we have placed this suggestion in section 8.2 as a limitation and future research direction of this paper, which is the red text **in Section 8.2 on page 31**.

We hope our explanations and modifications can meet your approval.

The references are as follows.

Muth JF. Rational Expectations and the Theory of Price Movements. Econometrica. 1961; 29(3): 315-335.

Su X. Intertemporal Pricing with Strategic Customer Behavior. Management Science. 2007; 53(5): 726-741.

Su X, Zhang F. Strategic Customer Behavior, Commitment, and Supply Chain Performance. Management Science. 2008; 54(10): 1759-1773.

Cachon GP, Swinney R. Purchasing, Pricing, and Quick Response in the Presence of Strategic Consumers. Management Science. 2008; 55(3): 497-511.

Shum S, Tong S, Xiao T. On the Impact of Uncertain Cost Reduction When Selling to Strategic Customers. Management Science. 2016; 63(3): 843-860.

Cao E, Luo H, Ma Y, Lü K. Dynamic pricing in the presence of strategic consumers with ‘experience-in-store-and-buy-online’. International Journal of Production Research. 2022; 61(20): 6873-6890.

Chen M, Chen Z. Recent Developments in Dynamic Pricing Research: Multiple Products, Competition, and Limited Demand Information. Production and Operations Management. 2015; 24(5): 704-731.

Famil A P, Seifi A. Dynamic pricing of differentiated products under competition with reference price effects using a neural network-based approach. Journal of Revenue and Pricing Management. 2024; 23: 575-587.

den Boer AV. Dynamic pricing and learning: Historical origins, current research, and new directions. Surveys in Operations Research and Management Science. 2015; 20: 1-18.

Cooper W L, Homem-de-Mello T, Kleywegt A J. Learning and Pricing with Models That Do Not Explicitly Incorporate Competition. Operations Research. 2015; 63(1): 86-103.

2. ***The*** ***literature review should be presented though in a clearer manner how the present study differentiates from existing research and makes a step towards better understanding the research problem. I recommend the authors to consult the following survey and empirical papers to contextualize your findings. This should help the readers to understand the novelty of your work (use the following references which are updated(recent) in the literature review section):***

- ***Dimitriadis, K. A., Koursaros, D., & Savva, C. S. (2025). Exploring the Dynamic Nexus of Traditional and Digital Assets in Inflationary Times: The Role of Safe Havens, Tech Stocks, and Cryptocurrencies. Economic Modelling, 107195.***
- ***Dimitriadis, K. A., Koursaros, D., & Savva, C. S. (2024). The influential impacts of international dynamic spillovers in forming investor preferences: a quantile-VAR and GDCC-GARCH perspective. Applied Economics, 1-21.***

**Our response:** Thank you for your comments. In the revised manuscript, we have referred to the writing style of the two references you recommended, and polished the differences and contributions of our paper to the literature. The specific modifications refer to the red text in **Section 2.3** on **pages 7-8**. We hope to meet your approval.

1. ***Also, the paper needs to improve the quality of the results section and how to present things (to be more explicit in discussing results, not so extensive,*** ***use the following article in the references section based on this: https://doi.org/10.1016/j.irfa.2024.103693).***

**Our response:** We appreciate your suggestion. In the revised manuscript, we referred to the writing form of the literature you recommended, and made corresponding modifications in the conclusion section. Please refer to the red text in **paragraph 1 of Section 8 on page 29, paragraphs 2-4 of Section 8.1 on page 29-30, and Section 8.2 on page 31.**

2. ***The authors should give more emphasis on the importance of their and should also be clearer in the conclusion section. Overall, this paper is interesting and well-structured, and it is my belief that it will be appealing for the readers of the journal. Furthermore, the abstract it would be better to be one whole paragraph which includes the most important result, the innovation of the research article and what does this article study.***

**Our response:** Thank you for your comments. In the revised version, we carefully read the literature you recommended and polished the importance of research issues and conclusions of the article. In addition, our abstract is presented in the form of one whole paragraph, which includes the research content, research methods, important conclusions. **Please refer to the red text in the Abstract on pages 1-2, paragraph 4 of Section 1 on page 3**. We hope our explanations and revisions can meet with your approval.

3. ***The paper should clearly address the following: What is the research question? Why is this question important and interesting? How did they approach the problem?*** ***and why is their empirical strategy persuasive?***

**Our response:** Thank you for your suggestions. Our response is as follows.

As for research questions, in the Section introduction, we first state the benefits and importance of implementing dynamic pricing with demand learning through literature and businesses examples (Fisher, 2009; Cheung et al., 2017; Naceva, 2024; Mailmodo, 2024; Dowling, 2023). Second, we emphasize the presence of strategic consumers and the necessity of implementing price guarantees based on dynamic pricing, as well as corresponding examples (e.g., JD.com, Dell (Williams, 2020), The Home Depot (Ritterman, 2021) and The Good Guys (Coates, 2022)). It is evident that dynamic pricing with price guarantee has gradually become a popular pricing strategy. However, it is not sure whether dynamic pricing with price guarantee always brings an increase in revenue, and how demand learning performs in dynamic pricing with price guarantee. Therefore, we have summarized four research questions for this article, which is shown in **paragraph 5 of Section 1 on pages 3-4 (the red text)**.

Regarding the importance of research questions, on the one hand, there is currently no research analyzing whether demand learning has superiority in dynamic pricing with price guarantees when facing strategic consumers. On the other hand, we believe that the questions have practical significance and can provide guidance for sellers' strategic choices.

For the research methodology used in this article, we illustrated the decision sequence in the text to state our research methods more intuitively, as detailed in the red text of **the third paragraph of Section 3.2 on pages 11-12.**

About the reason why empirical strategies are persuasive, our numerical results obtained through theoretical analysis present meaningful guidance for sellers. The consistency of these conclusions based on numerical results with empirical strategies in business practices indicates that they are reasonable and effective. **Please refer to Tables 5, 6, and 7 in the manuscript on pages 26-27, as well as the last two paragraphs of Section 6.2 on pages 27-28.**

We hope the above explanations and modifications can meet your approval.

The references are as follows.

Fisher M. OR FORUM-Rocket Science Retailing: The 2006 Philip McCord Morse Lecture. Operations Research. 2009; 57(3): 527-540.

Cheung WC, Simchi-Levi D, Wang H. Technical Note—Dynamic Pricing and Demand Learning with Limited Price Experimentation. Operations Research. 2017; 65(6): 1722-1731.

Naceva N. The Ultimate Guide to Amazon Dynamic Pricing Strategy in 2024 [Internet]. 2024 May 15 [Cited 2024 Sep 22]. Available from: <https://influencermarketinghub.com/amazon-dynamic-pricing/>.

Mailmodo. 6 Dynamic Pricing Examples From Different Industries [Internet]. 2024 May 22 [Cited 2024 Sep 22]. Available from: <https://www.mailmodo.com/guides/dynamic-pricing-examples/>.

Dowling L. The Future of E-commerce: Dynamic Pricing Strategies Powered by AI [Internet]. 2023 Oct 2 [Cited 2024 Sep 22]. Available from: <https://pathmonk.com/e-ommerce-dynamic-pricing-strategies-powered-by-ai/>.

Williams G. 10 Retailers That Offer Price Matching [Internet]. 2020 Nov 18 [Cited 2024 Sep 22]. Available from: <https://money.usnews.com/money/personal-finance/spending/articles/what-is-price-matching>.

Ritterman L. Home Depot Price Match & Price Adjustment Policy [Internet]. 2021 Dec 27 [Cited 2024 Sep 22]. Available from: <https://pricematchguarantee.net/home-depot-price-match/>.

Coates M. Get your Good Guys Price Match now [Internet]! 2022 Nov 16 [Cited 2024 Sep 22]. Available from: <https://thechampagnemile.com.au/good-guys-price-match-guide/>.

4. ***Does the paper clearly justify when demand learning improves revenue and when it actually hurts revenue, especially under a price guarantee?***

**Our response:** Thank you for your comments. In the revised manuscript, we justified when demand learning improves revenue and when it hurts revenue under two dynamic pricing alternatives, which is shown in **Observation 1 of Section 5.1 on pages 22-23 and Table 4 of Section 5.2 on page 24 (the red text)**. We hope our response meets with your approval.

5. ***Are the assumptions about consumer behavior (uniform valuations, common knowledge, rational expectations) realistic enough for the conclusions to be valid in real markets?***

**Our response:** Thank you for your constructive suggestion. In the revised manuscript, we recognize the limitations of the assumptions about consumer behavior in our paper and propose that bounded rational consumers can be considered in future research. The corresponding modifications are **the red text in section 8.2 on page 31**, and we hope these modifications meet with your approval.

6. ***How robust are the numerical results to changes in key parameters like inventory level, demand uncertainty, and consumer patience (δ)?***

**Our response:** Thank you very much for your comment. We have carefully examined the numerical results in the manuscript, and the specific conclusions are as follows.

1. When we conduct numerical results on the impact of initial estimate and prior market size uncertainty on demand learning performance, varying inventory levels $Q$, their influence trends on demand learning performance are consistent. Specific details are the red text in **Figures 2 and 3 of Section 5.1 on page 22**.

2. In the trade-off between strategy $DL$ and $GL$, when the prior market size uncertainty $CV$ is the main parameter, even if inventory level $Q$ or consumer strategic level $\delta$ changes, the trend of the percentage improvement of revenue generated by strategy $GL$ is similar. When inventory level $Q$ or consumer strategic level $\delta$ is the main parameter, even if the other two parameters change, the trend of the percentage improvement of revenue generated by strategy $GL$ is consistent. Please refer to the red text in **Tables 5, 6, and 7 of Sections 6.1-6.2 on pages 26-27**.

3. In the numerical results of inventory as a decision variable in section 7, while consumer strategic level $\delta$ is the main parameter, with varying the unit cost of products $c$ or the prior market size uncertainty $CV$, the trends of the numerical results remain consistent. In addition, if the unit cost of products $c$ or the prior market size uncertainty $CV$ is the main parameter, the numerical results maintain a similar trend when the other two parameters change. Please refer to the **Tables B1, B2, and B3 in Appendix B on S1 Appendix**.

Therefore, we believe that the numerical results are robust. I hope our response will receive your approval.

7. ***Do the authors convincingly explain how a seller should choose between “with price guarantee” and “without price guarantee” strategies at different product life-cycle stages?***

**Our response:** Thanks for your comments. In the revised manuscript, we analyzed the reasons behind the seller making trade-offs between Strategy $DL$ (dynamic pricing without price guarantee and with demand learning) and $GL$ (dynamic pricing with price guarantee and demand learning), as detailed in the **last two paragraphs of Section 6.2 on pages 27-28** (the red text). I hope our explanation will receive your approval.

**Review comments from reviewer 2**

**Major concerns:**

1. ***The manuscript makes a valuable contribution by addressing a clearly identified and underexplored research gap, as effectively summarized in Table 1. The simultaneous integration of demand learning, strategic consumer behavior, and price guarantees is a significant and timely endeavor that adds considerable depth to the existing literature.***

**Our response:** We greatly appreciate your recognition of the format in Table 1. Your approval has been incredibly encouraging and motivating.

2. ***A key model assumption—that sellers and consumers share a common prior and homogeneously update their beliefs via Bayesian learning (Section 3.3)—deserves further consideration. While this is a common and useful theoretical simplification, it may not fully capture real-world consumer behavior. Consumers often operate with limited information and may rely on heuristic decision-making rather than sophisticated Bayesian updates. I recommend that the authors explicitly acknowledge this as a limitation in the dedicated section and briefly discuss how incorporating heterogeneous priors or bounded rationality could influence their findings. This would not only strengthen the paper's self-awareness but also provide a compelling direction for future research.***

**Our response:** Thanks for your professional comments. In the revised manuscript, taking your suggestions into consideration, we proposed to further relax consumers into bounded rational consumers and to combine it with our models as a future research direction. The specific modifications **are in the red text of Section 8.2 on page 31**. We hope our modifications are approved by you.

3. ***While the numerical results are presented clearly as percentage changes, the economic significance of these differences could be strengthened. For instance, it is unclear whether a 1-2% revenue improvement would justify the operational shift to a new pricing strategy in practice. To enhance the impact of these findings, the authors could briefly contextualize the results by discussing the absolute revenue implications or commenting on the practical significance of the reported percentage changes from a managerial perspective.***

**Our response:** Thank you for your comments. Our results are presented in percentage, which is similar to Sen and Zhang (2009) and Aviv et al. (2019), Lin (2005), Talebian et al. (2014) and Shum et al. (2016). Besides, we believe that a 1% increase in revenue can significantly increase profits. Specifically, Feldman (1990) reports that in the airline industry, an increase of 1% in revenue is equivalent to 60% increase in profit; and Robinson (2021) states a 1% increase in revenue, profits increase by 14.29%. Moreover, In the numerical results of Section 6, only a small amount of $|\boldsymbol{k|}$ is less than 2%, most of $|\boldsymbol{k|}$ exceed 2%, and the highest is 113%, indicating that changing the pricing strategy can increase the expected revenue by more than double the original. Therefore, we believe that it is necessary and worth to justify pricing strategies in most cases. Finally, based on your suggestion, we have briefly explained the practical significance of revenue percentage changes from a managerial perspective. Please refer to **the red text in the third paragraph of section 6.2 on page 27** for specific modifications.

The references are as follows.

Feldman JM. Fares: To raise or not to raise. Air Transport World. 1990; 27 (6): 58-59.

Robinson J. Which is better: a 1% increase in price or a 1% improvement in your revenue growth rate [Internet]? 2021 June 30 [Cited 2025 Nov 27]. Available from: https://publications.pricingsociety.com/which-is-better-a-1-increase-in-price-or-a-1-improvement-in-your-revenue-growth-rate/.

4. ***There appears to be an inconsistency in the definition of the performance metric k in Section 6. The text defines it as a function of revenue (k = R_GL / R_DL - 1), which is the appropriate measure for strategy comparison. However, the headers and notes for Tables 4, 5, and 6 refer to it as a function of price (p_GL / p_DL - 1). This seems to be a typographical error. I recommend standardizing this to the revenue-based definition throughout the manuscript to avoid confusion.***

**Our response:** We appreciate your feedback. In the revised version, we have replaced the indicator $k=\frac{R^{GL}}{R^{DL}}-1$ in the notes of Tables 5, 6, and 7 with $k={R^{GL}}/{R^{DL}}-1$, and we bolded them for readers to better browse the manuscript. Corresponding revisions are seen the red text in Tables 5, 6, and 7 of **Sections 6.1-6.2** on **pages 26-27**.

5. ***The manuscript is generally well-written. To further enhance its clarity and academic tone, a careful proofread to polish minor*** ***grammatical points is recommended. For example:***

***(i) "demand learning brings*** ***slight benefits" could be refined to "demand learning*** ***yields only marginal benefits".***

***(ii) "is counterproductive for offering a price guarantee" could be rephrased to "can be counterproductive when a price guarantee is in place" or "******undermines the effectiveness of a price guarantee".***

**Our response:** We appreciate your valuable suggestions. Following your advice, we carefully proofread the entire manuscript, corrected any errors, and polished the grammatical points. We have also made corresponding modifications to the two examples you identified, which can be found **in the abstract on page 1, paragraph 2 of section 1.1 on page 4 and paragraph 2 of section 8.1 on pages 29-30**.

6. ***As a summary suggestion, I strongly advise the authors to incorporate a discussion on the limitation of the homogeneous, rational consumer belief assumption into Section 8.2 (Limitations and future research). Elaborating on this point will significantly improve the manuscript's rigor and scope for future work.***

**Our response:** Thanks for your comments. In the revised manuscript, following your suggestions, we emphasize the combination of bounded rational consumers and our models as a future research direction, exploring the differences in research results. Please refer to **the red text of Section 8.2 on page 31**. We hope our modifications meet your approval.

**Review comments about AE**

**Major concerns:**

***Please give attention to the comments made by reviewer II. I understand if all recommended changes are not feasible (might require additional fundamental research and restructuring): in such cases, please explain why certain comments were not addressed well.***

**Our response:** Thank you for your comments. In the revised manuscript, we have provided point-to-point responses to the reviewers’ comments and suggestions. We hope these revisions can meet your approval.
